# Purification and Characterization of Aporphine Alkaloids from Leaves of *Nelumbo nucifera* Gaertn and Their Effects on Glucose Consumption in 3T3-L1 Adipocytes

**DOI:** 10.3390/ijms15033481

**Published:** 2014-02-26

**Authors:** Chengjun Ma, Jinjun Wang, Hongmei Chu, Xiaoxiao Zhang, Zhenhua Wang, Honglun Wang, Gang Li

**Affiliations:** 1 School of Life Science, Yantai University, 30 Qinquan Road, Yantai 264005, China; E-Mails: chengjun-ma@163.com (C.M.); wangjinjun19890522@163.com (J.W.); mfsaih67@163.com (X.Z.); zhenhuawang@tom.com (Z.W.); 2 School of Pharmacy, Yantai University, 30 Qinquan Road, Yantai 264005, China; E-Mail: chu_hong_mei@sina.com; 3 Key Laboratory of Tibetan Medicine Research, Northwest Institute of Plateau Biology, Chinese Academy of Sciences, Xining 810001, China; E-Mail: hlwang@nwipb.cas.cn

**Keywords:** *Nelumbo nucifera* Gaertn, aporphine alkaloids, high-speed counter-current chromatography (HSCCC), isolation, 3T3-L1 adipocytes, glucose consumption

## Abstract

Aporphine alkaloids from the leaves of *Nelumbo nucifera* Gaertn are substances of great interest because of their important pharmacological activities, particularly anti-diabetic, anti-obesity, anti-hyperlipidemic, anti-oxidant, and anti-HIV’s activities. In order to produce large amounts of pure alkaloid for research purposes, a novel method using high-speed counter-current chromatography (HSCCC) was developed. Without any initial cleanup steps, four main aporphine alkaloids, including 2-hydroxy-1-methoxyaporphine, pronuciferine, nuciferine and roemerine were successfully purified from the crude extract by HSCCC in one step. The separation was performed with a simple two-phase solvent system composed of *n*-hexane-ethyl acetate-methanol-acetonitrile-water (5:3:3:2.5:5, *v*/*v*/*v*/*v*/*v*). In each operation, 100 mg crude extracts was separated and yielded 6.3 mg of 2-hydroxy-1-methoxyaporphine (95.1% purity), 1.1 mg of pronuciferine (96.8% purity), 8.5 mg of nuciferine (98.9% purity), and 2.7 mg of roemerine (97.4%) respectively. The chemical structure of four aporphine alkaloids are identified by means of electrospray ionization MS (ESI-MS) and nuclear magnetic resonance (NMR) analysis. Moreover, the effects of four separated aporphine alkaloids on insulin-stimulated glucose consumption were examined in 3T3-L1 adipocytes. The results showed that 2-hydroxy-1-methoxyaporphine and pronuciferine increased the glucose consumption significantly as rosiglitazone did.

## 1. Introduction

*Nelumbo nucifera* Gaertn, commonly called lotus, is a perennial aquatic crop grown and consumed over the world, especially in China, India, Japan, Korea, South East Asia, Russia and some countries in Africa [1]. All parts of this plant, such as fruits, seeds, stamens, roots and leaves, have been used not only as a vegetable and food garnish but also as a Chinese medicine and has been compiled in Chinese Pharmacopoeia [2]. Therefore, lotus is widely cultivated and consumed as food and medicine in China. In traditional Chinese medicine, lotus leaves are used to treat hematemesis, epistaxis, hemoptysis, hematuria, hyperlipidemia and obesity [3]. Lotus leaves are also used for tea and are commercially available in China. Pharmacologies studies revealed that its major constituents are aporphine alkaloids [4–7]. In the past, many studies have been focused on the isolation and pharmacology of its alkaloids components.

**Figure 1.**
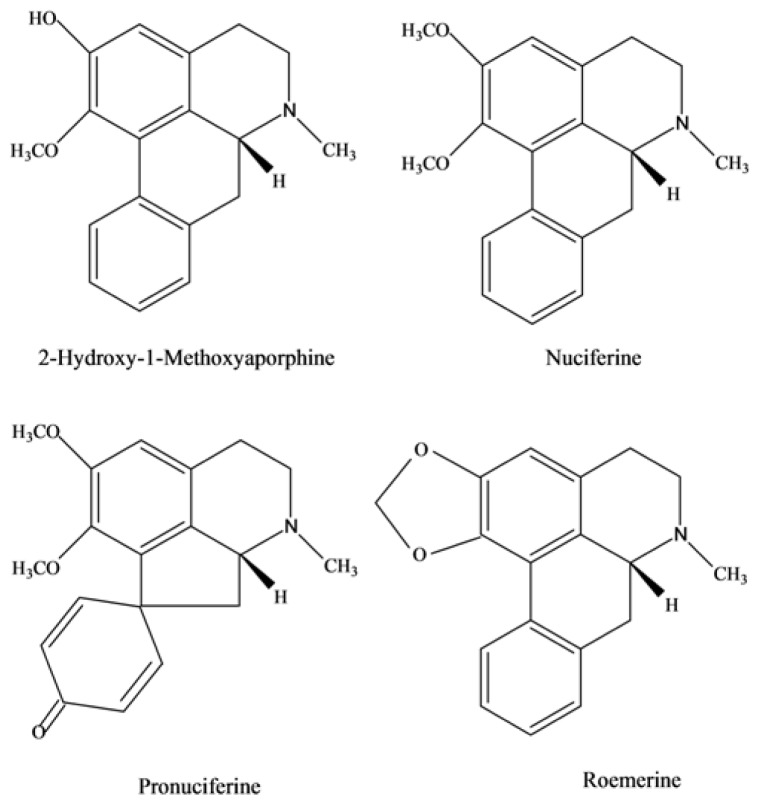
Structural formulas of aporphine alkaloids from leaves of *N. nucifera*.

Nuciferine and its analogues, 2-hydroxy-1-methoxyaporphine, pronuciferine, and roemerine (Figure 1), are four main aporphine alkaloids components of leaves of *N. nucifera*. Since the first isolation of nuciferine in 1962, these alkaloids have received considerable attention because of their reputation of chemical and biological activities including anti-HIV [8], anti-hyperlipidemic [9], anti-platelet [10], hypotensive [11], anti-oxidant [12], anti-microbial [13], anti-obesity [14,15], anti-diabetic [16], and melanogenesis inhibitory [3]. However, their pharmacological studies often suffer from the limits of sample purity and sources. To obtain pure compounds by conventional separation methods, such as using organic solvents to extract and column chromatography including silica gel, polyamide column, thin-layer chromatography (TLC), Sephadex LH-20 chromatography and high-performance liquid chromatography (HPLC) to isolate, have been used to isolate and purify aporphine alkaloids from leaves of *N. nucifera* [17–19]. However, the conventional extract and purify methods are tedious and time-consuming, requiring multiple steps, in which column chromatography requires a long time and a large volume of organic solvent, and worse still the sample are adsorbed onto the stationary phase irreversibly. As the alternative, a new technique, high-speed counter-current chromatography (HSCCC) is widely used to separate bioactive compounds from natural resources. It is a unique liquid-liquid partition chromatography technique that uses no solid support matrix so the adsorbing effects on stationary phase material and artifact formation, tailing of solute peaks and contamination can be eliminated. This technique has the maximum capacity with an excellent sample recovery and wider range of selection of solvent systems as compared to HPLC. Furthermore, it permits introduction of crude sample directly into the hollow column [20]. Table 1 summarized the difference of HSCCC and traditional methods to produce large amounts of pure alkaloid. Up to now, over 120 different alkaloid compounds from more than 30 plant species have been successfully separated [21].

It was reported that separation of three alkaloids (*N*-nornuciferine, nuciferine and roemerine) from lotus leaves was attempted via conventional HSCCC and pH-zone-refining CCC [22], but in this study, the actual separation larger quantities of mixture by HSCCC was failed mainly due to the poor retention of the stationary phase and resulted in a poor resolution. In addition, pH-zone-refining CCC is very confusing, and thus, various parameters of the mobile phase such as solvents, pH, and fraction ranges had to be adjusted. We have performed simple and efficient purification of four main aporphine alkaloids in lotus leaves including 2-hydroxy-1-methoxyaporphine, pronuciferine, nuciferine, and roemerine by HSCCC using an optimized two-phase solvent system. These purified alkaloids were analyzed by HPLC and chemical structures were identified by means of ESI-MS and NMR analysis. Further, insulin-stimulated glucose consumption of four separated aporphine alkaloids was measured by 3T3-L1 adipocytes.

## 2. Results and Discussion

### 2.1. Selection of Suitable Two-Phase Solvent System for HSCCC

Suitable two-phase solvent system is the key factor for a successful HSCCC separation, which should satisfy the following three main requirements: (a) the partition coefficient (*K*) value of the targeted compounds should be in the range of 0.5–2.0; (b) the two-phase solvents should be nearly equal volumes for each phase; and (c) the two-phase solvents should be a volatile solvent system [20,23]. In the study, the *K* values of four alkaloids were determined in the following four solvent systems: chloroform-methanol-water, ethyl acetate-methanol-water, *n*-hexane-ethyl acetate-methanol-water, and *n*-hexane-ethyl acetate-methanol-acetonitrile-water each at various volumes (Table 2). The result indicated that the solvent systems composed of chloroform-methanol-water had a lower *K* values (*K <* 0.5) and the systems composed of ethyl acetate-methanol-water had higher *K* values (*K >* 2.0). These solvent compositions did not prove satisfactory as an HSCCC solvent system for our isolation and separation. When the basally solvent system composed of *n*-hexane-ethyl acetate-methanol-water was used as the two-phase solvent system, as shown in Table 1, all *K* values of four targeted compounds were observably improved. Based on the system, different ratios of acetonitrile were added to adjust each *K* value to appropriate level. From the above, the two-phase solvent system composed of *n*-hexane-ethyl acetate-methanol-acetonitrile-water at a ratio of 5:3:3:2.5:5 (*v*/*v*/*v*/*v*/*v*) was found to be the best. Under the optimized separation conditions, the isolation of four target compounds was achieved with good resolution and the retention of the stationary phase are satisfactory (57.5%).

### 2.2. HSCCC Purification and HPLC Identification

**Figure 2.**
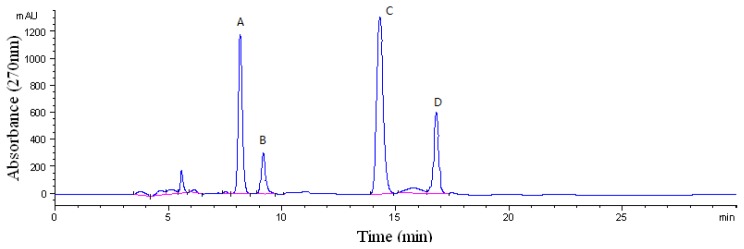
High-performance liquid chromatography (HPLC) chromatogram of the crude extract from the leaves of *N. nucifera*. Sample: Column: Discovery C18 column (25 cm × 4.6 mm, 5 μm); mobile phase: acetonitrile-water-triethylamine-glacial acetic acid (55:44:1:0.15, *v*/*v*/*v*/*v*); flow rate: 1.0 mL/min; detection wavelength: 270 nm. A: 2-hydroxy-1-methoxyaporphine; B: pronuciferine; C: nuciferine; D: roemerine.

**Figure 3.**
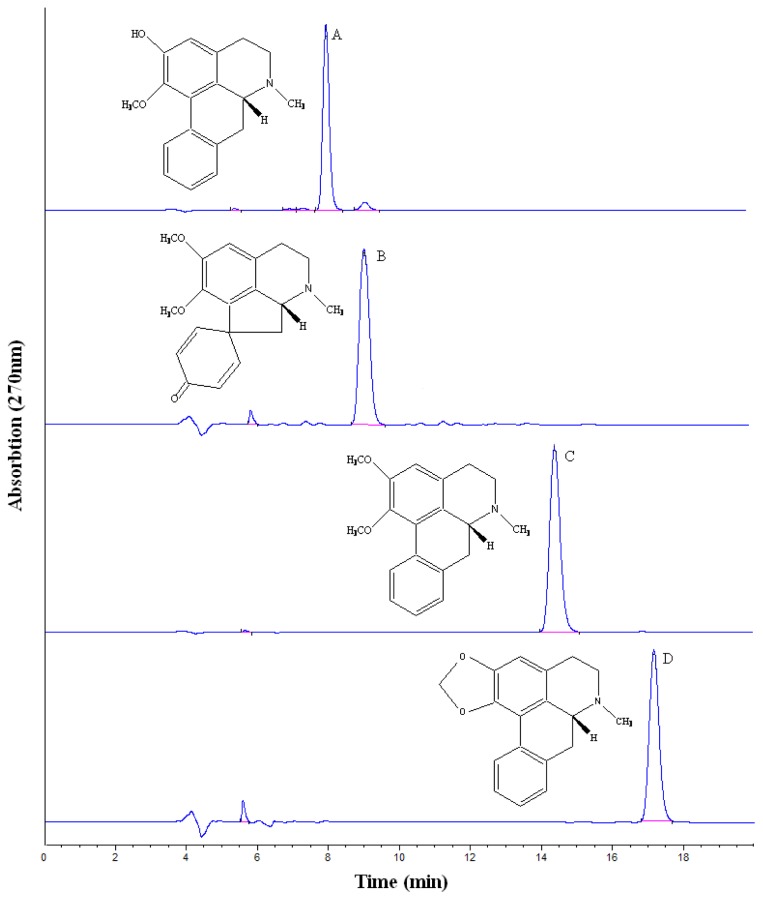
HPLC analyses of the fractions obtained by HSCCC. HPLC analysis conditions are the same as shown in Figure 2. A: 2-hydroxy-1-methoxyaporphine; B: pronuciferine; C: nuciferine; D: roemerine.

**Figure 4.**
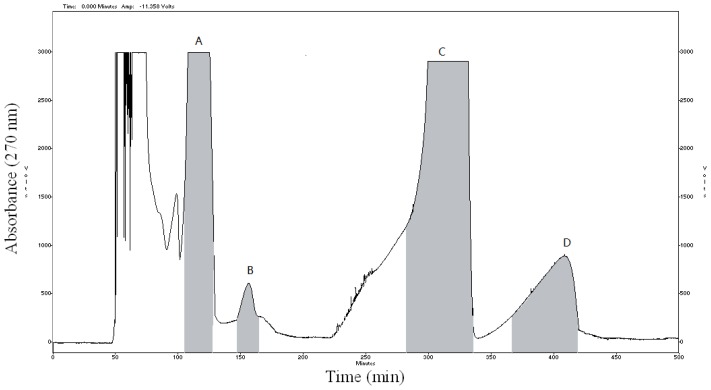
HSCCC chromatogram of crude extract from Lotus Leaf. Conditions: column: multilayer coil of 1.8 mm i.d.; polytetrafluoroethylene tube with a total capacity of 280 mL; rotary speed: 850 rpm; two-phase solvent system: *n*-hexane-ethyl acetate-methanol-acetonitrile-water (5:3:3:2.5:5, *v*/*v*/*v*/*v*/*v*); flow-rate: 2.0 mL/min; detection wavelength: 270 nm; sample size: 100 mg; injection volume: 12 mL; retention of stationary phase: 57.5%. Peaks: A: 2-hydroxy-1-methoxyaporphine; B: pronuciferine; C: nuciferine; D: roemerine.

The crude sample (100 mg) was dissolved in 12 mL mixture solution of upper phase and lower phase (1:1, *v*/*v*). The sample solution was separated and purified by HSCCC according to the procedure described. The retention of the stationary phase was 57.5%, and the total separation time was 500 min. Figure 2 shows HSCCC separation of the crude extract sample, along with the HPLC chromatogram analysis of purified compounds are shown in Figure 3. Based on the HPLC analysis and the elution curve of the HSCCC (Figure 4), all collected fractions were combined into different pooled fractions. A total amount of 6.3 mg of 2-hydroxy-1-methoxyaporphine (95.1% purity), 1.1 mg of pronuciferine (96.8% purity), 8.5 mg of nuciferine (98.9% purity), and 2.7 mg of roemerine (97.4%) were yielded in one-step, respectively.

### 2.3. Chemical Structure Identification

The structural identification of 2-hydroxy-1-methoxyaporphine, pronuciferine, nuciferine, and roemerine was carried out by electrospray ionization mass spectrometry (ESI-MS), ^1^H NMR and ^13^C NMR spectra as follows.

Fraction A: ESI-MS (*m*/*z*): 282 [M + H]^+; 1^H NMR (CDCl_3_, 400 MHz) *δ*: 8.26 (H, d, *J* = 8.0 Hz, OH), 7.21~6.68 (3H, m, H-8, 9,10), 3.66 (3H, s, O–CH_3_), 2.57 (3H, s, N–CH_3_), 2.45 (1H, d, *J* = 4.0, 8.0 Hz, H-6a); ^13^C NMR (100 MHz, CDCl_3_), *δ*: 143.3 (C-1), 126.4 (C-1a), 127.8 (C-1b), 146.5 (C-2), 114.0 (C-3), 28.7 (C-4), 128.1 (C-4a), 53.5 (C-5), 43.8 (N-CH_3_), 61.7 (C-6a), 33.9 (C-7), 136.1 (C-7a), 128.1 (C-8), 126.4 (C-9), 125.8 (C-10), 127.3 (C-11), 131.8 (C-11a), 60.5 (OCH_3_).

Fraction B: ESI-MS (*m*/*z*): 312 [M + H]^+; 1^H NMR (CDCl_3_, 400 MHz) *δ*: 8.32 (1H, d, *J* = 8.0 Hz, H-12), 7.14~7.25 (3H, m, H-8, 9,11), 6.79 (1H, s, H-3), 3.85 (3H, C_1_–OCH_3_), 3.60 (3H, C_2_–OCH_3_), 2.61 (3H, N–CH_3_); ^13^C NMR (100 MHz, CDCl_3_), *δ*: 146.2 (C-1), 137.9 (C-1a), 150.9 (C-2), 112.1 (C-3), 29.7 (C-4), 131.1 (C-4a), 54.8 (C-5), 43.9 (N–CH_3_), 31.5 (C-6a), 38.9(C-7), 130.6 (C-7a), 128.1 (C-8), 128.9 (C-9), 206.6 (C-10), 128.3 (C-11), 126.3 (C-12), 56.7 (OCH_3_), 60.3 (OCH_3_).

Fraction C: ESI-MS (*m*/*z*): 296 [M + H]^+; 1^H NMR (CDCl_3_, 400 MHz) *δ*: 8.36 (1H, d, *J* = 8.0 Hz, H-11), 7.20~7.33 (3 H, m, H-8, 9,10), 6.66 (1H, s, H-3), 3.89 (3H, s, C_1_–OCH_3_), 3.66 (3H, s, C_2_–OCH_3_), 2.59 (3H, s, N–CH_3_); ^13^C NMR (100 MHz, CDCl_3_), *δ*: 145.8 (C-1), 126.9 (C-1a), 151.0 (C-2), 111.3 (C-3), 29.1 (C-4), 128.3 (C-4a), 53.9 (C-5), 43.8 (N-CH_3_), 62.2 (C-6a), 35.5 (C-7), 135.6 (C-7a), 127.4 (C-8), 126.6 (C-9), 126.9 (C-10), 128.2 (C-11), 132.5 (C-11a), 55.3 (OCH_3_), 60.6 (OCH_3_).

Fraction D: ESI-MS (*m*/*z*): 280 [M + H]^+; 1^H NMR (CDCl_3_, 400 MHz) *δ*: 8.06 (1H, d, *J* = 8.0 Hz, H-11), 7.22~7.34 (3 H, m, H-8, 9,10), 6.56 (1H, s, H-3), 6.06 (1H, s, –CH_2_O–), 5.93 (1H, s, –CH_2_O–), 2.56 (3H, s, N–CH_3_), 2.44 (1H, s, H-6a); ^13^C NMR (100 MHz, CDCl_3_), *δ*: 142.6 (C-1), 116.4 (C-1a), 146.7 (C-2), 107.6 (C-3), 29.7 (C-4), 127.1 (C-4a), 53.7(C-5), 43.1 (N–CH_3_), 62.5 (C-6a), 34.9 (C-7), 135.2 (C-7a), 127.8 (C-8), 126.9 (C-9), 126.5 (C-10), 127.0 (C-11), 131.1 (C-11a), 100.6 (–CH_2_O–).

Compared with the date given in literatures [6,22,24,25], peak A–D in Figure 1 corresponded to 2-hydroxy-1-methoxyaporphine, pronuciferine, nuciferine and roemerine.

### 2.4. Insulin-Stimulated Glucose Consumption of 3T3-L1 Cell

**Figure 5.**
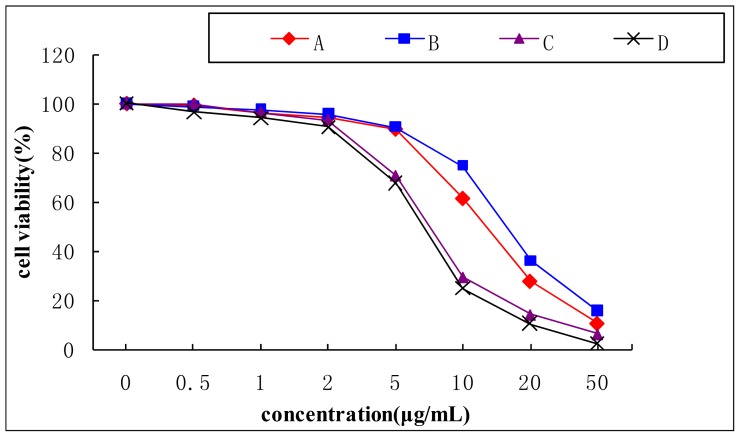
Cytotoxicity effect of aporphine alkaloids on 3T3-L1 cells. A: 2-hydroxy-1-methoxyaporphine; B: pronuciferine; C: nuciferine; D: roemerine.

**Figure 6.**
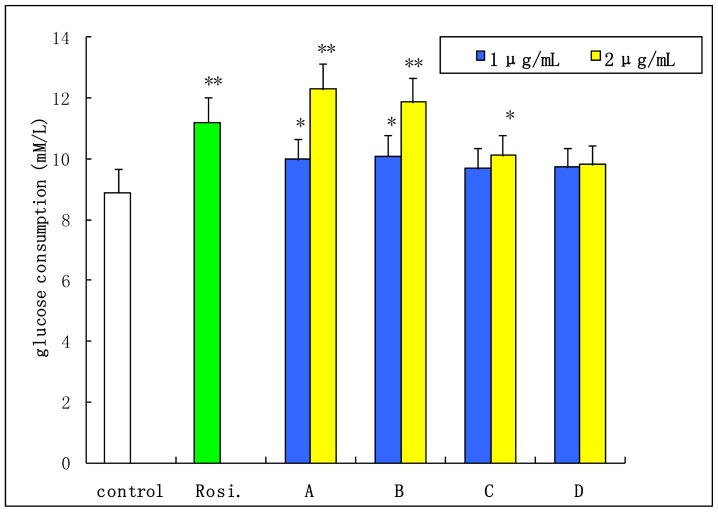
Effects of aporphine alkaloids on glucose consumption in differentiated 3T3-L1 adipocytes. The glucose consumption effect of 1 μmol/L rosiglitazone (Rosi.) as a positive control (Rosi. *vs*. control, *p* < 0.01). Data are the mean ± SD, *n* = 3. * *p* < 0.05; ** *p* < 0.01 *vs*. control group. A: 2-hydroxy-1-methoxyaporphine; B: pronuciferine; C: nuciferine; D: roemerine.

Figure 5 showed the cytotoxicity of aporphine alkaloids (0.5 to 50 μg/mL) when incubated with 3T3-L1 cells for 24 h. The survival cell was more than 95% when the compounds concentration was 2 μg/mL. When pre-adipocytes differentiate into adipocytes, the morphological alterations are induced by the presence of oil droplets in the cytoplasm; it also leads to simultaneously increase in both glucose uptake and lipid accumulation [26,27]. As shown in Figure 6, all the four compounds showed the effects of improving insulin-stimulated glucose consumption in differentiated 3T3-L1 adipocytes compared with the control group. Especially, 2-hydroxy-1-methoxyaporphine (A) and pronuciferine (B) exhibited the most potent glucose consumption-stimulatory activity at the concentration of 2 μg/mL. The glucose consumption were 12.29 and 11.88 mmol/L respectively, which were similar with the 11.2 mmol/L of the positive control (1 μmol/L rosiglitazone). It has been found that an analogue of these four aporphine alkaloid, possesses variable beneficial bioactivities including antioxidation in diabetic rats [28], up-regulating the adiponectin expression in 3T3-L1 cells [29] and improvement the endothelial function in spontaneously hypertensive rats [30]. Moreover, the adiponectin could upregulate the glucose uptake in adiopocytes through the activation of AMP-activated protein kinase (AMPK) [31]. These suggest that the AMPK signal pathway might mediate the glucose consumption of the two aporphine alkaloids. Moreover, the effects of 2-hydroxy-1-methoxyaporphine (A) and pronuciferine (B) on glucose consumption might be due to their strong polarity as shown in Figure 3.

## 3. Experimental Section

### 3.1. Chemical and Reagents

The leaves of *N. nucifera* were purchased from Yantai Chinese drug store (Yantai, China). Chloroform, *n*-hexane, ethyl acetate, and methanol were obtained from Yantai huada reagent Co. Inc. (Yantai, China). HPLC-grade methanol, acetonitrile were obtained from Tedia Company Inc. (Fairfield, CA, USA). Reverse osmosis Milli-Q water (18 MΩ) (Bedford, MA, USA) was used for all solutions and dilution. 3T3-L1 cells were obtained from the cell bank of the Institute of Biochemistry and Cell Biology (Shanghai, China). Phosphate buffered saline (PBS), DMEM medium; calf serum (CS), fetal bovine serum (FBS), penicillin-streptomycin solution and trypsin-EDTA solution were purchased from GIBCO Co. Inc. (Beijing Representative Office, Beijing, China). 3-(4,5-dimethylthiazol-2-yl)-2,5-diphenyltetrazolium bromide (MTT) and Oil Red O were purchased from Sigma-Aldrich Co. Inc. (Milwaukee, WI, USA).

### 3.2. Apparatus

HSCCC was performed using a Model TBE-300A HSCCC system (Shanghai Tauto Biotech Co., Ltd., Shanghai, China), equipped with a 280 mL coil column made of polytetrafluoroethylene tubing (I.D. of the tubing = 1.8 mm). The β-value of the preparative column varied from 0.42 at the internal layer to 0.63 at the external layer (β = *r*/*R*, where *r* is the distance from the coil to the holder shaft, and *R* is the revolution radius or the distance between the holder axis and central axis of centrifuge. For this apparatus, the revolution radius is 130 mm). The revolution speed of the apparatus could be adjusted in a range between 0 and 900 rpm. The solvent was pumped into the column by a Model S 1007 constant-flow pump (Beijing Shengyitong Technology Development Co., Ltd., Beijing, China), and continuously delivered by 270 nm absorption with a Model TSP 1000 UV detector (TSP, Waltham, MA, USA), the data was displayed and analyzed simultaneously on a Model Anastar 2.0 chromatographic data workstation (Tianjin Autoscience Instrument Co., Ltd., Tianjin, China). A manual injection valve with a 20 mL loop was used to introduce the sample into the column.

The analytical HPLC system used throughout this study was Agilent 1100 HPLC system including G 1311A pump, G1315B UV-vis detector and Agilent HPLC workstation (Agilent Technologies, Waldbronn, Germany), with a reversed-phase Discovery C18 column (25 cm × 4.6 mm I.D., 5 μm; Sigma-Aldrich, Bellefonte, PA, USA).

### 3.3. Preparation of Crude Extract from the Leaves of *N. nucifera*

The leaves of *N. nucifera* were dried at 60 °C and pulverized. 300 g of sample were extracted three times with 0.1 mol/L hydrochloric (3000 mL × 3) under 40,000 Hz of ultrasonic for 20 min each time. All extracting solution was combined, filtrated and 0.1 mol/L sodium hydrate was added into the filtrated until the pH of the solution reached 8.5, filtrated by filter paper. Then, the solutions were evaporated under reduced pressure and 60 °C to dryness, which yielded 2.1 g of crude alkaloid extracts for subsequent HSCCC separation.

### 3.4. Selection of Two-Phase Solvent Systems and Sample Solution for HSCCC

A number of two-phase solvent systems were tested by changing the volume of the solvent to obtain the optimum composition that gave suitable partition coefficient (*K*) values. The partition coefficient values were determined according to the literature [23,32]. In brief, approximately 4.0 mg sample of crude extracts were weighed in a 10 mL test tube to which 3 mL of each phase of the pre-equilibrated two-phase solvent system was added. After the tube was shaken vigorously for 1 min, the solution was quietly separated for a moment. Then, the upper and lower phases were analyzed by HPLC to obtain the partition coefficient (*K*) of four targeted compounds respectively. The *K* value was calculated as follows:

$$K=\frac{\text{HPLC peak}\;\text{area of solute in upper phase}}{\text{HPLC peak area of solute in lower}\;\text{phase}}$$

The selected solvent system (*n*-hexane-ethyl acetate-methanol-acetonitrile-water) was thoroughly equilibrated by repeatedly vigorously shaking in a separation funnel at room temperature. Two phases were separated shortly and degassed before use. The volume ratio of the five solvents is 5:3:3:2.5:5 (*v*/*v*/*v*/*v*/*v*). The upper phase was used as the stationary phase, while the lower phase was used as the mobile phase.

The sample solutions were prepared by dissolving the crude extract in the mixture solution of upper phase and lower phase (1:1, *v*/*v*) of the solvent system used for HSCCC separation.

### 3.5. HSCCC Separation Procedure

In each separation, the multiplayer-coil column was first entirely filled with the upper phase (stationary phase). The lower phase (mobile phase) was then pumped into the head end of the inlet column at a flow rate of 2.0 mL/min, while the apparatus was rotated at 850 rpm. After reaching hydrodynamic equilibrium, as indicated by a clear mobile phase eluting at the tail outlet, the sample solution (100 mg of the crude extract in 12 mL of the total volume of both phase that is 1:1) was injected into the column through the sample port. The effluent from the tail end of the column was continuously monitored with a UV detector at 270 nm as stated earlier and the chromatogram was recorded. Each peak fraction was collected manually according to the elution profile and determined by HPLC.

### 3.6. HPLC Analysis and Identification of HSCCC Fractions

The mobile phase was acetonitrile-water-triethylamine-glacial acetic acid (55:44:1:0.15, *v*/*v*/*v*/*v*) and the flow rate was 1.0 mL/min. A sample volume of 20 μL was injected. The crude sample and peak fractions obtained by HSCCC were analyzed by HPLC. The identification of HSCCC peak fractions was carried out by MS on mass spectrometer and by ^1^H NMR and ^13^C NMR spectra.

### 3.7. 3T3-L1 Cell Culture

T3-L1 cells were cultured in DMEM medium with 10% calf serum (CS), 1.5 g/L of sodium bicarbonate and 1% penicillin-streptomycin solution. Cells were cultured at 37 °C in a humidified atmosphere containing 5% CO_2_ and medium was replaced every other day until confluent.

### 3.8. Cytotoxicity Study of Aporphine Alkaloids

*In vitro* cell viability was measured using the MTT assay. Briefly, following the treatment of cells in 96-well plate (4 × 10^4^ cells/well) with different concentrations of aporphine alkaloids in DMEM containing 10% FBS for 20 h, the cells were incubated with MTT solution (0.5 mg/mL) for 4 h at 37 °C. The formation of a violet precipitate formazan was monitored at a wavelength of 490 nm with a Bio-Rad 550 microplate reader (Hercules, CA, USA).

### 3.9. Insulin-Stimulated Glucose Consumption Study

Insulin resistance was induced in 3T3-L1 cells as reference described with minor modifications [27]. Briefly, 2 day-post-confluent preadipocytes (day 0) were cultured in 10% FBS/DMEM medium containing 0.5 mmol/L isobutylmethylxanthine (IBMX), 1 μmol/L dexamethasone and 10 μg/mL insulin for 2 days. The cells were further incubated in the growth medium containing 1 μg/mL insulin for additional 2 days, and thereafter medium was replaced with fresh growth media, which was changed every 2 days thereafter until the cells were fully differentiated. Over 95% of the preadipocytes differentiated into adipocytes by day 8, as determined by Oil Red O staining. Then, the 3T3-L1 adipocytes were plated in 96-well plates in DMEM containing 10% FBS and 1 μmol/L dexamethasone. The tested compounds were added for an additional 2 days. The glucose content in culture medium was measured by glucose oxidase method and glucose consumption was calculated.

## 4. Conclusions

In conclusion, our present finding is the first report on separation and purification of four aporphine alkaloids including 2-hydroxy-1-methoxyaporphine, pronuciferine, nuciferine, and roemerine from lotus leaves by HSCCC using the new two-phase solvent system on one step. Through the insulin-resistance 3T3-L1 adipocytes, we evaluated the glucose consumption-stimulatory activity of purified compounds. The overall results of the present study indicate that HSCCC is a powerful technique in separating and purifying bioactive compounds from natural sources. All the four compounds possessed concentration-dependent cytotoxicity in 3T3-L1 cells in which 2-hydroxy-1-methoxyaporphine and pronuciferine powerfully enhanced the glucose consumption in adipocytes differentiated from 3T3-L1 cells as rosiglitazone did. These finding may be benefit for the popular use of lotus leaves in blood sugar balance and weight loss in China.

## Figures and Tables

**Table 1.** The compare of high-speed counter-current chromatography (HSCCC) and the traditional methods.

Category	HSCCC	Traditional Methods
Partition method	liquid-liquid partition	solid-liquid partition
Separation time	time-saving	tedious and time-consuming
Separation process	one step	multiple steps
Dosage of organic solvent	smaller volume	larger volume
Sample adsorbed loss	smaller	larger
Selection range of solvent systems	wider	limited
Sample condition	crude sample	multiple steps pre-treatment sample

**Table 2.** The *K* (partition coefficient) values of four alkaloids in different solvents systems.

Two-Phase Solvents	Partition Coefficient (*K*)				

	Ratio (*v*/*v*)	A	B	C	D
chloroform/methanol/water	10/0/10	0	0	0	0.01
	10/1/9	0	0	0.01	0.09
	10/3/7	0.02	0.05	0.10	0.19
	10/5/5	0.07	0.14	0.25	0.38
	10/6/4	0.04	0.17	0.21	0.29
	10/7/3	0.02	0.15	0.18	0.20
ethyl acetate/methanol/water	10/1/9	10.46	9.53	16.93	22.50
	10/4/6	3.38	4.08	7.08	15.67
	10/6/4	2.05	3.91	4.74	8.21
	10/8/3	1.71	3.01	3.89	7.08
*n*-hexane/ethyl acetate/methanol/water	5/5/5/5	1.01	1.19	1.60	2.85
	5/3/3/5	0.94	1.41	2.02	3.90
	5/2/2/5	0.80	1.88	2.91	4.32
	5/1/1/5	1.28	2.69	3.76	6.54
*n*-hexane/ethyl acetate/methanol/acetonitrile/water	5/3/3/0.5/5	0.89	1.22	1.74	3.16
	5/3/3/1.0/5	0.77	1.03	1.59	2.44
	5/3/3/1.5/5	0.69	0.89	1.42	2.17
	5/3/3/2.0/5	0.56	0.77	1.25	1.94
	5/3/3/2.5/5	0.51	0.76	1.20	1.87
	5/3/3/3.0/5	0.48	0.66	1.22	1.79

Expressed as: A: 2-hydroxy-1-methoxyaporphine; B: pronuciferine; C: nuciferine; D: roemerine.
